# Simulated Photovoltaic Solar Panels Alter the Seed Bank Survival of Two Desert Annual Plant Species

**DOI:** 10.3390/plants9091125

**Published:** 2020-08-31

**Authors:** Rebecca R. Hernandez, Karen E. Tanner, Sophia Haji, Ingrid M. Parker, Bruce M. Pavlik, Kara A. Moore-O’Leary

**Affiliations:** 1 Department of Land, Air & Water Resources, University of California, Davis, One Shields Avenue, Davis, CA 95616, USA; 2 Wild Energy Initiative, John Muir Institute of the Environment, University of California, Davis, One Shields Avenue, Davis, CA 95616, USA; 3 Ecology and Evolutionary Biology Department, University of California, Santa Cruz, 1156 High St, Santa Cruz, CA 95064, USA; karen.e.tanner@gmail.com (K.E.T.); sohaji@ucsc.edu (S.H.); imparker@ucsc.edu (I.M.P.); 4 Conservation Department, Red Butte Garden and Arboretum, University of Utah, Salt Lake City, UT 84108, USA; bruce.pavlik@redbutte.utah.edu; 5 U.S. Fish and Wildlife Service, Pacific Southwest Region, 3020 State University Drive East, Sacramento, CA 95819, USA; kara_moore-oleary@fws.gov

**Keywords:** disturbance, drylands, photovoltaic, plant community, plant traits, rare species, renewable energy, seed traits, seed banks, solar energy

## Abstract

Seed bank survival underpins plant population persistence but studies on seed bank trait-environment interactions are few. Changes in environmental conditions relevant to seed banks occur in desert ecosystems owing to solar energy development. We developed a conceptual model of seed bank survival to complement methodologies using in-situ seed bank packets. Using this framework, we quantified the seed bank survival of two closely related annual desert plant species, one rare (*Eriophyllum mohavense*) and one common (*Eriophyllum wallacei*), and the seed bank–environment interactions of these two species in the Mojave Desert within a system that emulates microhabitat variation associated with solar energy development. We tracked 4860 seeds buried across 540 seed packets and found, averaged across both species, that seed bank survival was 21% and 6% for the first and second growing seasons, respectively. After two growing seasons, the rare annual had a significantly greater seed bank survival (10%) than the common annual (2%). Seed bank survival across both species was significantly greater in shade (10%) microhabitats compared to runoff (5%) and control microhabitats (3%). Our study proffers insight into this early life-stage across rare and common congeners and their environmental interactions using a novel conceptual framework for seed bank survival.

## 1. Introduction

Understanding seed banks has proven to be exceptionally challenging, rendering our knowledge of early life history traits incomplete across the plant kingdom [1,2]. Studies are needed to enumerate and predict changes in seed banks, particularly to ascertain if plant species’ or communities’ seed banks respond to environmental changes [2,3,4,5,6,7,8]. Studies of annual seed banks in deserts may be especially useful to study seed bank trait–environment interactions because these environments are characterized by extreme variability in temperature and precipitation [9,10]—variability that increasingly characterizes Earth’s environments. Additionally, many deserts are subject to rapid land-cover changes owing to renewable energy development, notably owing to ground-mounted photovoltaic (PV) solar energy [11,12,13]. Although seed bank longevity and composition have been examined in deserts around the world [14,15,16,17,18], empirical in situ studies of seed bank trait-environment interactions are few in the context of deserts and, indeed, across all ecosystems ([2,4,5] but see [19,20,21]).

Seeds produced from an individual plant often aggregate locally and become buried in soils over time, forming a seed bank. Seeds within the bank embody a diversity of ages, densities, and traits, including traits that drive plant survival and fitness. In natural environments, different plant species may co-occur within a single plant community, including common and rare species. Studies of seed banks of rare and common species in desert annual plant communities may aid an understanding of seed bank traits. For several reasons, desert annual plants are an established model system for seed bank studies: their aboveground life cycle stages are exceptionally fast, they are mostly surficial in the soil (≤2 cm depth), and their responses to changes in the environment often occur over short durations of time [8,22,23,24,25,26].

Despite the population- and community-level significance of seed banks, seed bank studies to date have focused primarily on a limited set of seed traits, predominantly seed size and mass [2,5]. Of those, only seed mass has been evaluated systematically despite increased evidence that germination-related seed traits, like survival, are better predictors of plant community dynamics than morphological seed traits, and that seed mass may be under- or over-estimated owing to low sample sizes [1,27,28].

Limitations in the empirical evaluation of seed banks stem from the logistical challenges of tracking seeds and their properties at and below the soil surface [29]. In natural environments, this accounting of seeds is certainly no easy feat. Seeds may decay owing to pathogenic or fungal interactions [19,30], be moved by anthropogenic and/or geophysical processes [21], consumed or buried by animals and/or litter [31], or die before germination even begins—processes that may take place over time scales ranging from hours to decades [19].

Seed bank survival is described as the duration (e.g., months) and/or degree (%) of viability of individual seeds and seed populations in known and in situ locations of the soil and/or soil profile and over precise and biologically relevant durations [2,5]. Seed bank survival, in all its manifestations to date, has also been named ‘longevity’ or ‘lifespan’ or ‘viability’ [5,28] and is sometimes not accurately differentiated from ‘seed bank persistence’—the latter defined as a functional characterization of the seed bank indirectly or in a relatively general sense, often without exactness or determining the viability of individual seeds. This is problematic as seeds may carry over, surviving through subsequent years, creating an age-structured seed bank [4,19]. This practice has led to conflicting, ambiguous, and even erroneous conclusions from seed bank studies [2,32]. Recent work has underscored the utility of methods that allow one to quantify seed bank survival using, for example, “buried bags” or “seed bank packets”, where in situ seeds can be exhumed over time conferring multiple observations [19,20,21]. A conceptual framework to standardize the accounting of seed bank survival using this method does not yet exist but may be useful [2,4,24,32].

Persistent seed banks (those in which seeds reside for >1 y) are typically found in environments with less predictable environmental characteristics (e.g., precipitation), like deserts [33,34], or where density-dependent effects prevail (e.g., herbivory, competition, [35]). Also, in deserts, high variability in the volume and frequency of rainfall drive selection for dormancy as a form of bet hedging that allows a fraction of seeds to remain ‘banked’ for germination in future, ostensibly more favorable years. The persistent seed bank may play a critical role in maintaining the survival and genetic variability of species across ecological levels, especially during periods of abiotic shifts. By ensuring the presence of annual seeds poised to take advantage of favorable conditions, this mechanism provides a critical foundation for desert food webs, as many desert species, including rodents and ants, are dependent on annual plants [31,33].

Fine-tuned environmental responsiveness of seed traits can elicit dramatic annual variation in aboveground abundance of taxonomic and functional groups, including across common and rare groups of species [36,37]. Some have posited that rare species may serve as pre-adapted replacement species within plant communities should climatic and/or environmental characteristics change, subsequently casting the ‘rare’ plant into a new role as ‘common’ [8,38]. Observations of rare species with relatively higher seed bank survival than common species within a single community may, in part, support this hypothesis. Differences in response to similar environmental conditions are seen aboveground between rare and common species of the diminutive annual *Eriophyllum* genus in the Mojave Desert (Tanner et al., in review [39]). Overall, elucidation of the seed bank survival of common and rare species provides a unique insight into the functional ecology of plants and the resilience of a plant community to changes in microhabitat conditions, and not just those from climate change.

In addition to land-use change owing to agriculture and urban expansion, deserts have been recently impacted by ground-mounted solar energy development [11,40,41,42,43,44,45]. Solar energy construction and its infrastructure comprised primarily of broad PV panels can facilitate direct and indirect disturbances to the microhabitats of seed banks, notably changes to light, surface temperature, and hydrological regimes. Global installed PV solar energy exceeded 500 GW of capacity at the end of 2018 and is projected to provide a majority of global energy reaching 30 to 70 terawatts by 2050, underscoring the importance of understanding its impact on terrestrial ecosystems [46]. The tilt of a panel effectively intercepts and diverts precipitation to its lower edge, creating an area of runoff which can be significantly greater in soil moisture than areas under the panel [47]. In shaded areas directly under a panel, Tanner et al. [47] found soil temperatures were significantly cooler and photosynthetically active radiation (PAR) lower. Such changes may elicit dramatic responses in seeds. For example, desert seeds in microhabitats with higher soil moisture may be more likely to germinate and/or be infected by fungal pathogens facilitated by such anomalously wet conditions [30]. Tanner et al. [47] and Grodsky and Hernandez [45] found significant alterations in aboveground plant community composition and structure owing to ground-mounted panel infrastructure but did not evaluate effects on seed banks [2]. As both theoretical and empirical studies have identified seed bank survival as a driver of annual plant demography in variable environments, it is important to understand how seed banks may respond to changes in the environment owing to the siting of solar energy infrastructure in places where desert annuals exist [13,48,49,50,51,52].

**Figure 1:**
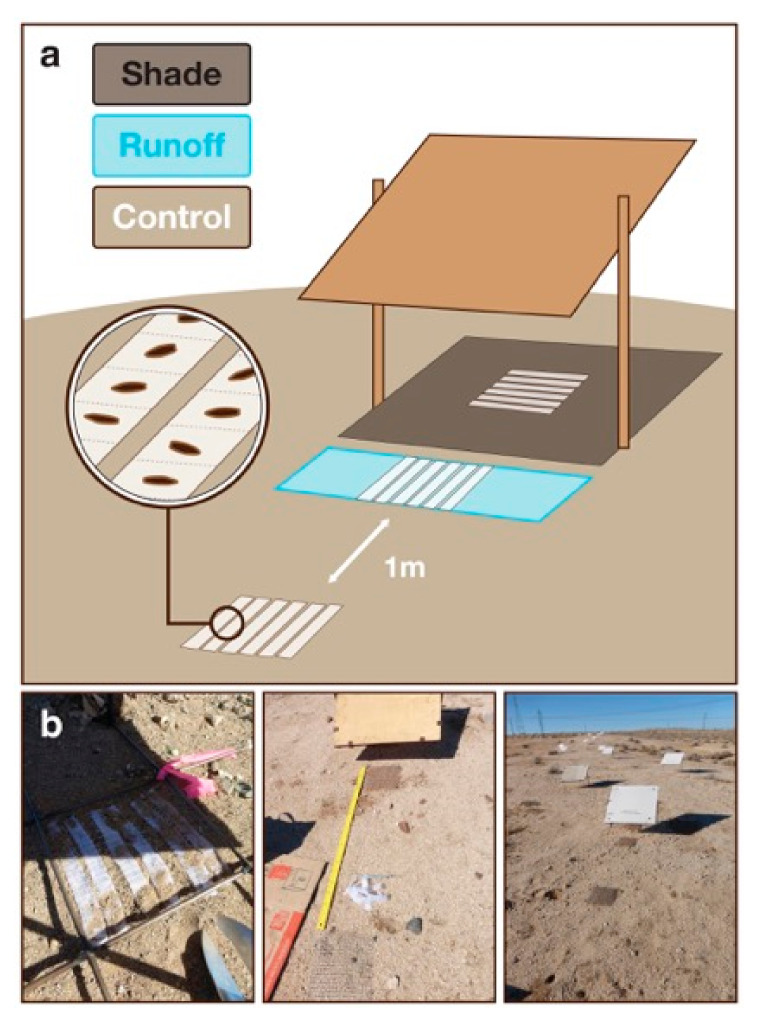
Seed bank packet and solar energy development microhabitats experimental design. (**a**) Graphical representation of the seed bank installation design. (**b**) Seed bank packet installation in fall 2016. Seed bank packets were deployed in Shade, Runoff, and Control microhabitats at each site (Caliche Pan site shown above).

**Figure 2:**
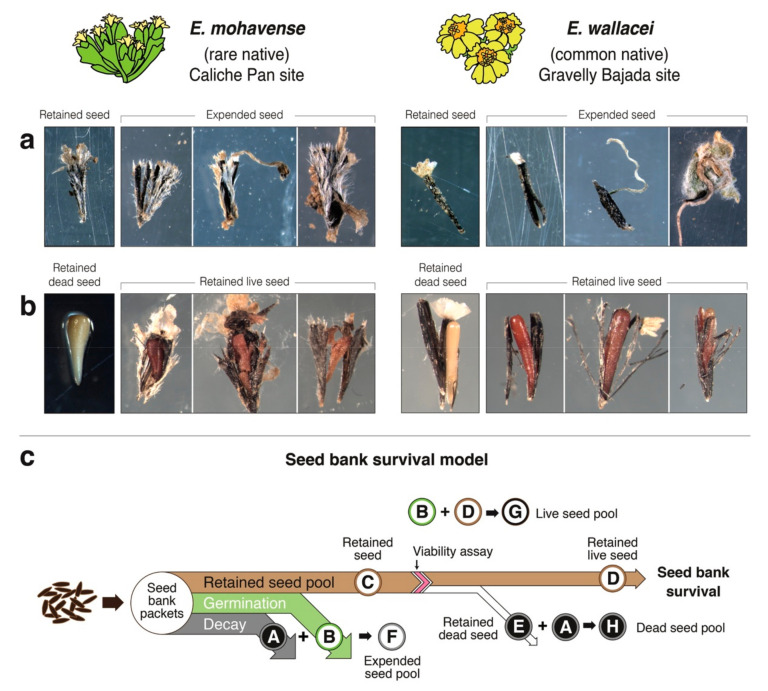
A seed bank survival conceptual model and photos of seed types in the seed bank; seed of both species are ~2 mm long. (**a**) Retained seed and expended seed (includes germinated and decayed seed) retrieved from the Caliche Pan site (left) and the Gravelly Bajada site (right). (**b**) Retained dead seed (no red tissue following tetrazolium stain), and retained live seed (tissue stained reddish brown) retrieved from the Caliche Pan site (left) and the Gravelly Bajada site (right). (**c**) Conceptual model of seed bank survival providing a quantitative visualization of seed bank seed types, pools, and survival. After deploying and collecting seeds from a seed bank packet, seeds are visually evaluated to differentiate and enumerate decayed seed (i.e., dead or dying seeds; grey flow, A) with split seed coats and no radicle, and germinated seed (green flow, B), with radicles or other plant tissue present. Added together, these values represent the expended seed pool (F) of the seed bank. The remaining seed represents the retained seed pool (brown flow, C). All retained seed is then tested for viability with a stain-based assay (e.g., with tetrazolium staining or similar methods). Retained dead seeds (white flow, E) are enumerated and sorted from retained live seed (final brown flow, D). The percentage of live seed (D) in the retained seed pool represents seed bank survival (%). Further, the sum of germinated and live seeds (B + D) are expressed collectively as the live seed pool (G). The sum of decayed seed and retained dead seed (A + E) can be expressed as the dead seed pool (H).

**Figure 3:**
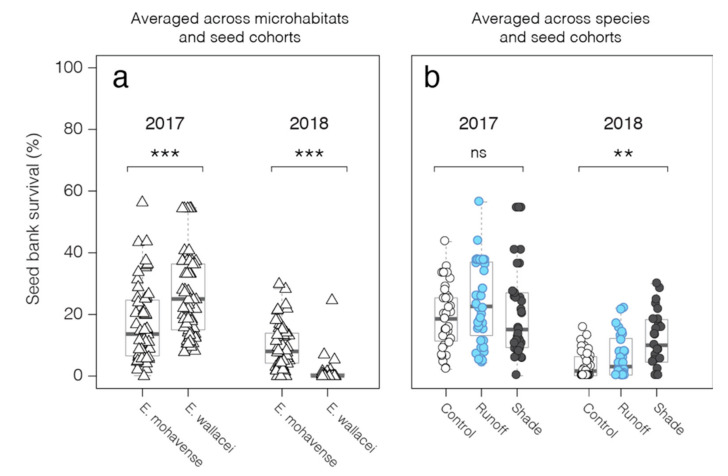
Seed bank survival (%) of *E. mohavense* and *E. wallacei*. (**a**) Seed bank survival (%) in 2017 and 2018, averaged across microhabitats and cohorts. (**b**) Seed bank survival by microhabitat in 2017 and 2018 (averaged across species and seed cohorts). ** Indicates a significant difference at the *p* < 0.01 level. *** Indicates a significant difference at the *p* < 0.001 level.

In this study, we evaluate the seed bank survival of two desert annual plant congeners, one rare and one common. These two closely related annual plant species, *Eriophyllum mohavense* ((I.M. Johnst.) Jeps.-rare, Barstow woolly sunflower) and *E. wallacei* ((A. Gray) A. Gray-common, Wallace’s woolly daisy), occur in the Western Mojave Desert, an area notable for ground-mounted solar energy development [43]. We use a seed bank packet method to quantify in situ seed bank survival directly (Figure 1) and present a complementary conceptual framework (Figure 2). We use this framework to compare seed bank survival between the common and rare annual desert plant species [8,38] and to evaluate seed bank survival across microhabitats (shade, runoff) and control microhabitats that vary in soil moisture and light and emulate effects of PV solar energy infrastructure (Figure 3). We interpret our findings both in the context of theory on rare and common species, including responses to changes in environmental conditions, and as they inform the study of annual plant responses to microhabitat variation needed to guide land-use planning and management decisions [47]. Overall, our aim is to contribute conceptual and observational insight into desert annual seed banks, including their responses to changes in microhabitats imposed by anthropogenic disturbances [5].

## 2. Results

### 2.1. Seed Bank Survival Conceptual Model

We present a conceptual model of seed bank survival (Figure 2c) to provide a framework to quantify and compare seed bank data from seed bank packets across various environments and through time. The model employs a Sankey diagram populated with data from seed bank packets to confer a quantitative and proportionally accurate visualization of seed bank seed types, pools, and ultimately, seed bank survival.

Our model defines the total number of seeds collected in all seed packets (Table 1, Supplementary Tables S1 and S2) within a cohort as the seed bank (100%; Figure 2c). After deploying and collecting seeds from a seed bank packet, we visually evaluated seeds to differentiate between decayed seed (grey flow, A) with split seed coats and no radicle (i.e., dead or dying), and germinated seed (green flow, B), with radicles or other plant tissue present. Added together, these values represent the expended seed pool (F) of the seed bank. The remaining seeds represent the retained seed pool (brown flow, C). This capacity observed in seed banks has also been referred to in past studies as seed bank retention, referring to both living and dead intact seed remaining in the soil (Tanner et al., in review [39]). Next, we tested retained seeds for viability (e.g., with tetrazolium staining or similar methods). Retained dead seeds (white flow, E) were sorted from retained live seeds (final brown flow, D). We defined the percentage of live seed (D) in the retained seed pool as seed bank survival (%). Further, we defined the sum of germinated and live seeds (B + D) as the live seed pool (G). We expressed the sum of decayed seed and retained dead seed (A + E) as the dead seed pool (H).

#### Seed Bank Survival

We found our seed bank conceptual model, using results from our seed bank packets, served as a useful framework to quantitatively evaluate and visualize seed bank survival of 3240 and 1620 seeds of *E. wallacei* and *E. mohavense*, respectively, in the Mojave Desert across three unique microhabitats (Figure 1, Table 1). In total, we sewed and tracked 4860 seeds across 540 seed packets in the Mojave Desert. Across both species, seed bank survival in control microhabitats averaged 19% in packets buried for one growing season, and 3% in packets buried for two growing seasons (Figure 3b).

### 2.2. Seed Bank of a Rare and Common Annual Plant Congener Pair

**Figure 4:**
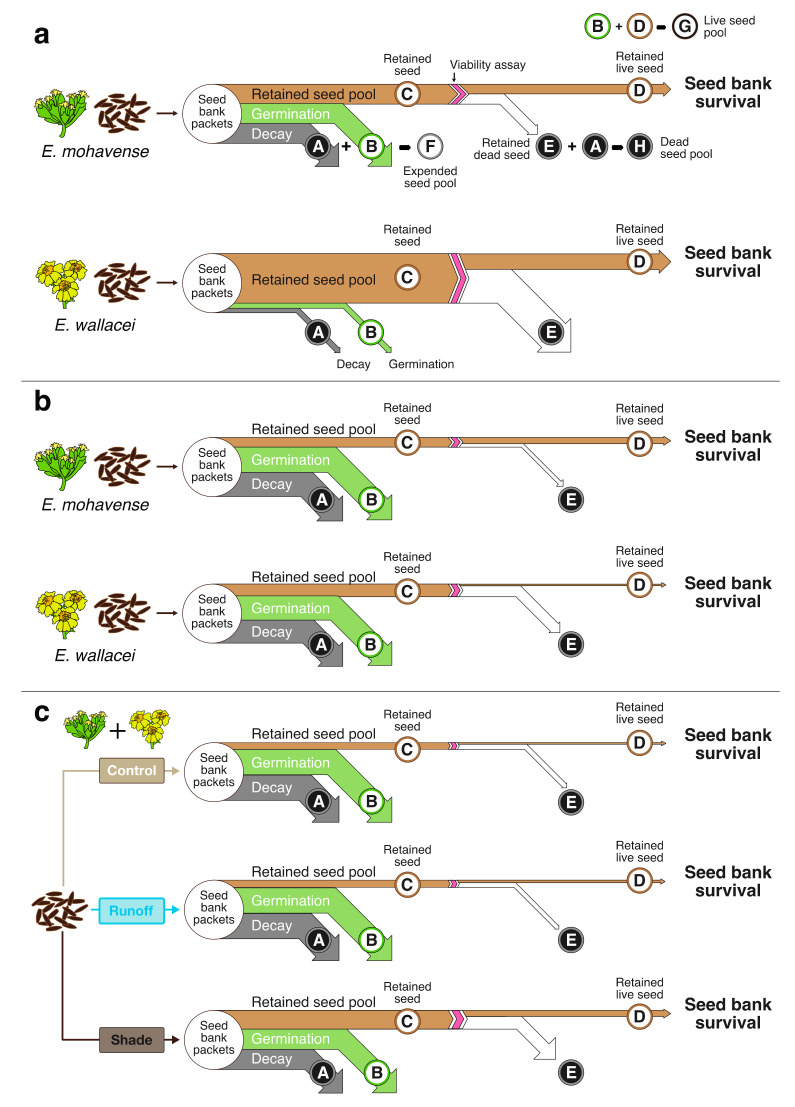
The seed bank survival model showing empirical seed bank pools and types for *E. mohavense* and *E. wallacei.* Models in (**a**) show flows for each species after one year of burial, with seed bank traits averaged across cohorts and microhabitats (we found no significant differences in retained seed across microhabitats; see top row of panels in Figure S1). Models in (**b**) show flows for each species after two years of burial, with seed bank traits averaged across both cohorts and all microhabitats. Models in (**c**) show flows for Control, Runoff, and Shade microhabitats averaged across both species and cohorts after two years of burial, when we observed higher seed retention in the Shade compared to the other two microhabitats (see bottom row of panels in Figure S1). We cannot confidently partition decayed seed (A) from germinated seed (B) in the expended seed pool (due to the delay between the winter annual germination period and collection of packets in spring), so we visualize these flows as equivalent in size. Flows exiting the staining assay (pink chevron) visualize the percentage of live seed for a subset of the retained seed pools (C) exposed to staining-based assays.

After one year, we found a greater number of expended (decayed or germinated) seeds of the rare species *E. mohavense* (67.3% on average across cohorts and microhabitats), compared to the common species, *E. wallacei* (17.3% across cohorts and microhabitats; Supplementary Figure S1, top row). Thus, the average retained seed pool for *E. wallacei* (82.7%, Supplementary Figure S1e,g) was significantly larger than the retained seed pool for *E. mohavense* (32.7%, Supplementary Figure S1a,c; Supplementary Table S2). Staining-based assays on a subset of the retained seed pool revealed a 49.0% seed staining rate for *E. mohavense* and a 31.1% seed staining rate for *E. wallacei* (Supplementary Figure S2, top row; Supplementary Table S3). Consequently, in the first growing season, we determined that seed bank survival (averaged across all cohorts and microhabitats) was significantly greater for *E. wallacei* (26.1%; for calculations, see Supplementary Table S4) than *E. mohavense* (16.2%; W= 2548, *p*-value = 0.00003577, Figure 4a). In control microhabitats, survival of *E. wallacei* (21.9%) was also significantly greater than *E. mohavense* (15.9%; W = 275, *p*-value = 0.04365).

After two years, the expended seed pool for *E. mohavense* and *E. wallacei* was 84.4% and 79.3%, respectively. Although a decline in retained seed over time was expected, this decline was much more dramatic for *E. wallacei* between the first and second year. For example, the retained seed pool for *E. wallacei* dropped from ~83% to ~21% (Supplementary Figure S1, compare e,g to f,h) in the second year of burial, while *E. mohavense* dropped from ~33% to ~16% (Supplementary Figure S1, compare Figure S1a,c to Figure S1b,d). The percentage of seed in retained seed pools for all microhabitats combined did not differ between the rare and common species after two years (estimated marginal mean contrast *p*-value = 0.2553; Supplementary Figure S1, bottom row).

Stain-based assays on a subset of seed retained through two years of burial yielded seed staining rates of 62.3% and 11.4% for *E. mohavense* and *E. wallacei*, respectively (Supplementary Figure S2, bottom row)-the lack of decline in *E. mohavense* seed staining rates suggest that survival remains high for aging retained seed. Next, we calculated seed bank survival and found that second-year seed bank survival was significantly greater for *E. mohavense* than *E. wallacei* in the control microhabitat (2018 in Figure 3a; 5.6% (rare) vs. 1.0% (common); W = 55, *p*-value = 0.0005517) and across all microhabitats (9.8% (rare) vs. 2.2% (common); W= 234, *p*-value < 0.00001).

### 2.3. Seed Bank-Environment Interactions

Microhabitat effects on seed bank traits, averaged across species, changed with time spent buried (Table 2 and Table 3). After the first growing season, seed bank survival (Equation (1)) did not significantly differ across control (18.9%), runoff (23.5%), and shade (21.7%) microhabitats (Supplementary Table S4b, chi-squared = 2.9228, df = 2, *p*-value = 0.2319). This result reflects a lack of microhabitat effects on seed retention and staining rate (indicating viability) in the first year of burial for both species and cohorts (Supplementary Figures S1 and S2, top panels).

Seed bank survival = pl × ps(1)

where pl = proportion of retained seeds (to total number of seeds in seed bank packets) and ps = seed staining rate, i.e., proportion of viable retained live seeds (to total number of seeds assayed).

In the second year, seed bank survival averaged across species (Supplementary Table S4b) was significantly greater in shade (9.7%) relative to the runoff (5.0%) and control (3.3%) microhabitats (Figure 3b, chi-squared = 12.651, df = 2, *p*-value = 0.00179). Averaged across cohorts, *E. mohavense* seed bank survival was 5.6% in the control microhabitat, 9.6% in the runoff, and 14.0% in the shade. For *E. wallacei* seed bank survival was 1.0% in the control, 0.3% in the runoff, and 5.3% in the shade. Hence, survival fell by 82.4% and 78.9% for control and runoff locations, but only 55.5% for seeds in shade locations. Microhabitat effects on seed bank survival in the second year were driven by effects on the retained seed pool. The retained seed pool from the shade microhabitat in the second year was larger than the pool in the control microhabitat, for both species and seed cohorts (Supplementary Figure S1, bottom panels). Seed staining rates were not affected by microhabitat in the second year for either species (Supplementary Figure S2, bottom panels).

## 3. Discussion

How long does a seed survive and what factors impact its survival? To date, results from efforts to quantify seed bank survival remain few [19,20,21]. Jiménez-Alfaro et al. [50] found that seed germination-related traits are deemphasized relative to morphological traits. Over 75% of the 226 experiments they assessed used morphological traits (e.g., seed mass) to study plant communities—despite increasing evidence that germination traits are better predictors of plant community dynamics than morphological ones [28]. While the study presented here focuses on the seed bank survival of just two species, it provides insight into annual desert seed banks, their response to changes in microhabitat associated with solar energy development, as well as the opportunities and challenges associated with studying seed bank survival.

Several authors have documented an inconsistency of seed bank survival terms across studies and this may, in part, contribute to challenges in the comparison of results across different studies [2,32,51]. While the challenges associated with comparing studies are many and will not be overcome in one fell swoop, we found our conceptual model of seed bank survival efficiently organized and conferred a unique visualization of results from seed bank packets. Specifically, it allowed us to empirically ‘follow’ a seed through this early life-stage: a seed is dispersed on or into the soil and may enter the ‘expended seed pool’ if it resides in a favorable condition for germination, thus becoming a ‘germinated’ seed type. Alternately, if it resides in an unfavorable condition, it may die and become a ‘decayed’ seed type. Alternatively, a seed may persist in situ, existing within the ‘retained seed pool.’ In this pool, it may die and become a ‘retained dead’ seed type, or carryover into subsequent growing season(s) as a ‘retained live’ seed type. If a seed stays in this pool for at least one growing season, it contributes to a persistent seed bank (versus a transient seed bank, enduring less than one year; [33]). We used a one-year timeline; however, if needed, our model may be applicable to other timelines for seeds exhibiting more complex dormancy cycles (e.g., seeds that are 1.5–2 years old before they can germinate; [52]).

Scientists employing demographic models to understand plant communities have struggled to integrate belowground life-stages often owing to a lack of data, instead focusing on more amenable aboveground traits (e.g., fecundity). However, Kalisz and McPeek [55] found that carryover of seeds in the seed bank is a key driver of population growth rate in a hybrid study (i.e., demographic modeling constrained by empirical observations of seed banks) of a winter annual. Data aligning with our seed bank framework can serve as inputs in demographic models, potentially greatly improving their realism and predictive capacity. In a complementary study, Tanner et al. [39] used the ‘retained seed pool’ and ‘seed bank survival’ data presented here to parameterize matrix models that revealed PV panel impacts on *Eriophyllum* demographic performance (e.g., shade suppressed population growth of *E. mohavense*).

The use of seed bank packets (or “bags”) may confer some advantages over other methods related to seed bank survival [4,19,20,21,29,56]. For example, direct age measurements of 53 viable seeds from a Sonoran Desert winter annual plant (*Pectocarya recurvata*) using ^14^C tandem accelerator mass spectrometry showed that age measurements had a 95% confidence interval of 2.3 y [4]. This method is problematic because it precludes the capacity to relate seed bank properties to even coarse, annual-scale environmental drivers (e.g., a “good rainfall year”) and also measured some seeds as being from “the future” (i.e., 1–3 years from the time of analysis). Further, all seeds in this study were viable, which is unrepresentative of real seed banks and sample size was low relative to our method: we tracked approximately 5000 seeds in seed bank packets. That said, we did face logistical challenges using seed bank packets, which confers an opportunity for advancement in future deployments. For example, we could not confidently partition decayed seed from germinated seed in the expended pool (due to the delay between the winter annual germination period and collection of packets in spring), and assessing pre-burial seed viability would provide valuable insight into mechanisms underlying differences in the expended seed pool between species. Seeds are also a critical food resource for desert granivores [25], but the fine weave of seed bags may function as a barrier to seed collection, especially by ants, and we were not able to design a complementary, simultaneous experimental method to quantify granivory of seeds in situ. Lastly, although some species (e.g., those with very small or large seeds) may not be conducive to chemical viability assays, alternative assays can be used. Chiquoine and Abella [51] used a combination of floating and pressure (by hand) to test seed viability across over 50 different species.

### 3.1. Simulated Photovoltaic Solar Panels and Seed Bank Survival

Studies of seed bank trait-environment interactions are needed to understand responses to anthropogenic disturbances and predict demographic and ecological consequences of such changes into the future, including changes in growth rates and extinction [7,19,20,21]. Deserts provide an ideal setting for experimentation given that relatively small variation in amounts of rainfall can elicit dramatic effects on the density and life history traits of desert annuals, including seed emergence and seed survival [57,58]. Studies to date are limited. For example, Pluntz et al. [56] characterized seed traits solely from presence/absence of aboveground flora using Hidden Markov modeling but assumed seed survival is constant over time, ruling out the potential for any environmental interactions with seeds. However, field-based studies show that microsite conditions, like drought [6] or removal of an invasive plant [51] can impact seed bank traits.

In general, we found that seed bank survival was significantly greater in shade microhabitats compared to runoff microhabitats and control microhabitats. Shade microhabitats within PV systems are notable for receiving less PAR and having lower soil moisture and temperatures, and we found this to be true for our experiment [47]. For example, soil moisture was consistently lowest in the shaded microhabitat after storms, and significantly so at the Caliche Pan site [47]. We found that Shade microhabitats significantly increased seed bank survival for both species after the second growing season owing to some combination of decreased soil moisture, soil temperature, and PAR. Our results also corroborate findings on the role of natural shade regimes as a driver of seed germination responses, for example, under the canopies of trees, shrubs, and cacti serving as nurse plants [59]. For example, Kos and Poschlod [60] found that shade facilitated and inhibited germination in plant species adapted to the shade of nurse plants and open interspaces, respectively, in the Kalahari savannah. Studies and restoration activities within solar energy installations may yield improved environmental outcomes (e.g., soil carbon sequestration) if species adaptation to shade and light conditions are considered and inform the spatial distribution of species mixes under panels (versus interspaces) [61,62].

Our findings suggest that drier conditions under panels may contribute to increased carryover, at least temporarily (2–3 years) after dispersal. We posit this because drier conditions slow seed decay in other systems, and this effect has been linked to reductions in soil pathogen activity [63,64]. We note that a limitation of our study is that we cannot separate effects of burial duration from differences in rainfall across the two years of our seed bank study. Nonetheless, higher seed bank survival is coupled with a lower amount of seeds germinating and/or decaying. Although we cannot tease apart environmental effects (i.e., soil temperature, moisture, light) under shade microhabitats, the altered conditions under PV panels are important. Within a ground-mounted solar energy facility, the shaded expanse is far larger relative to cumulative runoff areas (R. Hernandez, pers. observ.), and thus it is reasonable to assume that a greater number of seeds are subject to changes in microhabitat by shading from PV panels than changes associated with areas of runoff. If seeds reside in the seed bank longer (i.e., higher seed bank survival) and ultimately do not germinate or die, management actions may be needed to supplement the number of seeds under shade to compensate for these effects. This should be further explored in considering mitigation and/or restoration under PV panels.

In the second year, we found no significant differences between seed bank survival in the runoff microhabitat (5.0%) compared to our control microhabitats (3.3%). This is in good standing with results at the same site from Tanner et al. [47], which showed no significant differences in soil moisture between runoff microhabitats and control microhabitats at the Caliche Pan or Gravelly Bajada site. This suggest that the seed banks may respond less dramatically to increased water inputs compared to the edaphic changes observed under panels, namely reduced soil moisture and temperature [47]. In a concurrent experiment, we evaluated effects of fungicidal treatment on seed fungal infection rates at the Caliche Pan and Gravelly Bajada sites, but did not find differences in infection rates across microhabitats at either site [65]. However, infection rates were much higher at the Caliche Pan site, consistent with the higher water holding capacity of soils at this site [47]. Future work is needed to understand the role that soil moisture plays in seed–fungal relationships as these relationships in deserts are understudied (see Li et al. [30]).

### 3.2. Seed Bank Survival of a Rare and Common Annual Plant Congener

Measurements of seed bank pools can be used to characterize the endurance of a seed bank (e.g., transient vs persistent; [33]), a key demographic trait. The earliest population models incorporating seed banks predicted that the long-term growth rate of a population is optimized with the evolution of delayed germination (i.e., carryover) but a “true annual life cycle” assumes the absence of a seed bank [48,66]. In our study, we found that seed bank survival in two desert congeners after the second growing season was 5.6% and 1.0% for *E. mohavense* and *E. wallacei*, respectively. Thus, we can conclude that both *Eriophyllum* species have, at least, a short-term persistent seed bank, which is consistent with other desert annual seed bank observations [33,34].

Germination-related traits were first documented by Grime et al. [67] and Keddy [68]. Seed bank survival, along with other seed traits (e.g., desiccation tolerance, light requirements, seed release height), are thought to regulate the nature and magnitude of processes that filter regional species pools into local plant communities [50]. Thus, it might be expected that geographically and/or phylogenetically close species would share similar seed bank traits; however, sympatric and closely related species often present divergent seed traits, including common and rare congeners.

More recently, differences in functional trait expression between common and rare annual plant species are posited to result from adaptations by rare species to historical and/or future climatic and environmental conditions [8,55,69,70]. Specifically, rare species may serve as pre-adapted replacement species (i.e., either maladapted and/or specialized) within plant communities. In other words, if and when climatic and/or environmental characteristics change, rare plants can become common thereby sustaining plant community persistence [8,38]. Our observation showing rare species with a relatively higher seed bank survival than its common congener species, suggests the validity of this hypothesis. However, a meta-analysis of rare and common seed bank survival datasets or an experimental design of equal rigor would be needed to adequately test this hypothesis. After two growing seasons, we found seed bank survival of the rare species was significantly greater in control microhabitats and also across all microhabitats. *E. mohavense* is not only rare but an edaphic specialist to soils high in calcium carbonate (i.e., an inorganic carbon-rich landform, sometimes called ‘caliche’ [47], which has recently been found to be less constant over geologic periods than previously thought and is dynamic across decades and centuries. In an annual plant community of the Sonoran Desert, Venable and Kimball [71] found that community diversity remained consistent across several decades but dramatic shifts in community composition were observed in which rare species became common and vice versa. Collectively, seed banks may support such shifts and affect community stability through time [5]. These results suggest that rare plants may be pre-adapted replacement species; however, we caution that this should be further explored and confirmed using these methods across larger functional and/or phylogenetic groups of common and rare plant congeners [8,38]. Regardless, these results suggest that higher seed bank survival of *E. mohavense* compared to *E. wallacei* may indicate greater reliance of the rare species on long-lived seed for persistence in its perhaps increasingly narrow niche [33].

We observed differences in the retained seed pool across seed cohorts, especially for the rare species, which might result from multiple, non-exclusive drivers (Supplementary Figure S1). Such patterns could emerge as a consequence of longer laboratory storage time for 2015 versus 2016 seed, but they could also arise from inherent differences in seed cohorts, possibly resulting from prevailing environmental conditions [72], maternal effects [73], quality of available pollination services, or other factors.

## 4. Materials and Methods

### 4.1. Study Sites

The Mojave Desert is a rain shadow desert (i.e., 124,000 km^2^) in the North American Southwest and one of the last remaining areas of intact wilderness in the contiguous United States [43]. Notably, it is characterized by diverse landforms and edaphic elements, including caliche pans (i.e., a hardened sedimentary layer of calcium carbonate), bajadas (i.e., a broad region along the base of a mountain front where alluvial fans coalesce), playas (‘dry lakes’), biological soil crusts, washes that serve as ephemeral stream drainages, desert pavement, and volcanic fields. Diversity of landforms and soil-based elements uniquely impact desert plant community composition and structure and thus are important to identify in seed bank studies (Tanner et al., in review [39,47,74,75]).

**Figure 5:**
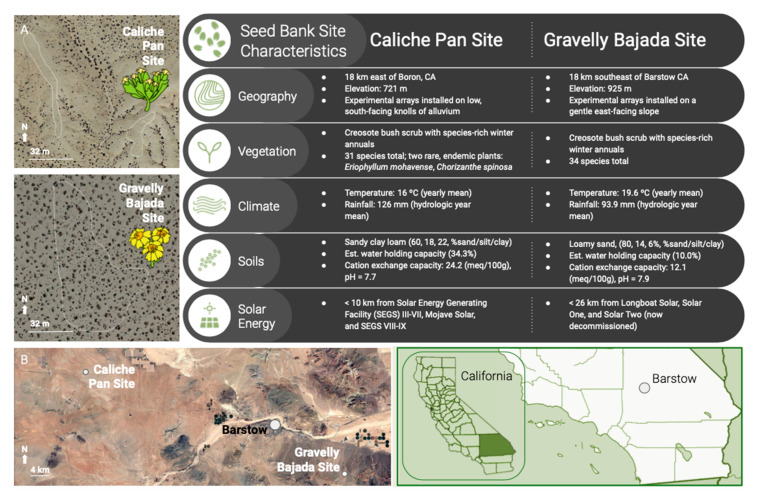
Site-level maps and characteristics of the Caliche Pan and Gravelly Bajada Sites (Western Mojave Desert, CA, USA; A. Google Earth, 222 m alt.; B. Landsat/Copernicus, 721 m alt.).

Our study focused on the Western Mojave Desert (7431 km^2^, CA, USA), a plant endemism hotspot [76,77]. Recently, the Western Mojave Desert has been identified as a distinct region where renewable energy development has been high relative to other areas of the Mojave [43]. Here, we identified two field sites, characterized by two common landforms: a Caliche Pan and Gravelly Bajada site (Figure 5). In good rainfall years, the Caliche Pan site supports high densities of *E. mohavense* and the Gravelly Bajada site supports high densities of *E. wallacei*. Both sites are low in elevation, topography, and slope and high in solar resources, emulating site characteristics of economically feasible ground-mounted solar energy development. Recent rainfall (2012–2018) was low at the Caliche Pan site, remaining below the historic 25th percentile in five of the seven years. Rainfall at the Gravelly Bajada site was below the 25th percentile in three of seven years and in 2017 was at the 89th percentile [47]. The sites are characterized as a creosote bush scrub plant community (<20% cover of perennials) and support species-rich annual plant communities in years of ample rainfall (approximately 15% of these species are shared across sites). A complete description and analysis of site characteristics are provided by Tanner et al. [47].

### 4.2. Artificial Photovoltaic Installation and Experimental Design

We constructed experimental panels with a fixed, 30° tilted collecting surface area of 0.37 m^2^, supported by metal frames and mounted approximately 0.2 m off the ground at the Caliche Pan and Gravelly Bajada sites. Each experimental panel represents one plot, composed of “shade,” “runoff,” and “control” microhabitats (Figure 1). The Shade and Runoff microhabitats are delineated by the ~60 × 62 cm shadow cast under the panels at solar noon and ~16 × 60 cm runoff area in front of the bottom edge of the panel closest to the soil surface, respectively. The control microhabitat was established one meter to the south of the runoff microhabitat. Thirty-two of these panels were installed in 2011 and were reallocated to this experiment in 2016, when we also installed four additional panels per site, for a total of twenty per site. We covered all panels with clear plastic sheeting (4 mm Coroplast, corrugatedplastics.net, Hillsborough, NJ, USA) in summer 2016 to emulate the smooth surface of a PV panel and facilitate rainfall runoff. Within sites, plots were selected to minimize the heterogeneity of substrate and slope. Due to patchy distribution of annual species in shrub interspaces, plot locations were chosen non-randomly to contain threshold numbers of focal species (*n* = 100 for the rare plant and *n* = 26 for the common plant, based on different population densities), ensuring habitat conditions suitable for seed germination. All plots were established in areas where they would not be shaded by nearby shrubs or the infrastructure associated with nearby plots.

### 4.3. Study Organisms

*Eriophyllum mohavense* (I.M. Johnst.) Jeps. (Barstow woolly sunflower) and *Eriophyllum wallacei* (A. Gray) A. Gray (Wallace’s woolly daisy) are small (1–2.5 cm and 1–15 cm, respectively), closely related winter annuals in the Asteraceae family. Both species germinate in fall or winter and set achenes (hereafter called seed) in late spring; seeds of both species are ~2 mm long [78] (Figure 2a). *Eriophyllum mohavense* is only found in creosote-bush scrub plant communities within the Mojave Desert and Desert Mountain geographic subdivisions. Here, it is found in small, isolated patches on edaphic islands in the western Mojave Desert [79]. *Eriophyllum wallacei* is a self-incompatible forb and common [80]. It occurs in the same vegetation type and subdivisions as *E. mohavense* but also across southwestern United States, Wyoming, and northwestern Mexico. Our focal taxa have comparable morphology and life history strategies, but while *E. wallacei* is widespread, *E. mohavense* is a rare California endemic designated as imperiled (NatureServe, 2018), with a California Rare Plant Rank 1B.2 [81] and listed species status under the Desert Renewable Energy Conservation Plan [82].

### 4.4. Seed Acquisition for Seed Bank Packets

In spring 2015 and 2016, we collected fruiting plants of *E. mohavense* and *E. wallacei* (*n* > 100 individuals per species and year) from plants growing in the open at each site. From these plants, we harvested mature seed that met our quality criteria: dark black in color, firm. The 2015 seed cohort was stored under ambient laboratory conditions at the University of California, Santa Cruz (UCSC) until 2016 collections were made the following spring. Appropriate storage conditions and duration are key methodological considerations of seed bank studies and best practices are necessary, especially when collecting seeds across multiple cohorts [83]. After-ripening of seed under summer conditions may also affect the dormancy and germination of winter annual species [29]. Here our primary aim was to test for differences in seed bank traits across microhabitats. All seeds were stored together in a dry lab at room temperature, so although delayed burial and lack of after-ripening may change germination behavior, it should not introduce biases across microhabitats.

### 4.5. Seed Bank Packet Construction

We sowed seed for each species independently into polyorganza fabric sleeves, creating sets of seed bank packets for each species and seed cohort (Figure 1a). Each packet was subdivided into cells containing a single seed, and the number of cells per packet was determined by the seed available for each cohort and species (Table 1). We distributed packets evenly among control, runoff, and shade microhabitats in fall 2016, and buried them under a layer of soil ~5 mm deep. Shallow burial depths provide the best opportunity for microhabitat differences to affect seed fates, and Freas and Kemp [84] found that desert annual seeds showed highest germination when buried 1–3 mm deep. Based on an earlier pilot experiment, we chose to increase this depth slightly to ensure that packets would remain covered during the long periods required by our study. Finally, packets were fixed in place with a square of ½” hardware cloth and 5” roofing nails (Figure 1b). On each subsequent visit, we checked that packets remained buried, and if any fabric was visible we added soil to achieve full coverage. This happened rarely and when it did occur only a small proportion of the packet was exposed. We collected the seed bank packets in March 2017 and 2018 (Supplementary Table S1) and stored packets in paper envelopes in ambient lab conditions at University of California, Santa Cruz (UCSC). Within three months of collection, we inspected seeds individually under a dissecting microscope at UCSC.

### 4.6. Staining Assays

Formal assays were carried out during summer on seed recovered from packets collected the previous spring, with one exception: resource constraints delayed assay of the 2016 cohort collected in spring 2017 until the summer of 2018. However, staining results for this cohort do not suggest that additional storage time negatively affected seeds. Specifically, we found no differences in staining rate for *E. mohavense* cohorts recovered in 2017, and observed a higher staining rate for the 2016 *E. wallacei* cohort recovered in 2017 (Supplementary Figure S2). Before formal assays, intact seeds were imbibed in deionoized water for 24 h. We prepared a 1% solution of 2,3,5-triphenyltetrazolium chloride and deionized water, and cut seeds longitudinally using a precision knife (Xacto #11 blade) to expose the embryo and pericarp. *E. wallacei* seeds were soaked in solution for 24 h at 17 °C, and *E. mohavense* seeds were soaked for 6 h at 35 °C. Within 1 h following soak, all exposed embryos were examined under a high-power stereoscope (SMZ800, Nikon Inc., Tokyo, Japan). The intensity and completeness of embryo staining varied among individuals as well as across species, so we classified seed according to the presence or absence of stain. Individuals with completely white embryos were considered retained dead seed, and those exhibiting any stain were considered retained live seed (Figure 2b). Effectiveness of seed viability assays may differ across species and thus similar methodological assessments should be performed to evaluate the accuracy of viability-based observations for individual plant species.

### 4.7. Seed Bank Survival Conceptual Model and Methods

First, we counted seeds with visible radicle material or compromised (e.g., split, empty hulled) seed coats. The time lag between typical germination periods for winter annuals and packet collection in spring meant that we could not be sure whether compromised seeds germinated and then decayed, or simply decayed. Thus, in this study, we added both of these seed types together and identified them as the expended seed pool (Figure 2c, flows A, B); however, these seed bank types could be enumerated with additional experimental effort, and this task would be easier for species that do not bet hedge (i.e., where germination is more predictable in time). All remaining intact seed represents the retained seed pool (Figure 2c, flow C; Supplementary Table S2).

As one cannot ascertain whether retained seeds are living visually, we performed tetrazolium staining as our viability assay (Figure 2c, see pink ‘chevron’) on a subset of the retained seed pool. For most species, observations of living seeds demonstrate that respiring tissues convert colorless tetrazolium chloride to a water-insoluble red carmine formazans, staining living tissue red [85]. Some authors caution this method may be less reliable for species exhibiting seed dormancy because truly dormant seeds may fail to stain [86]. We tested this technique for our species, boiling seed (*n* = 10 seeds per species) in water for 5 min prior to staining. All boiled embryos remained white, confirming dead tissue will not stain. We next conducted trials to identify optimal heating and soaking times for live seed of each species. *E. mohavense* stained slowly in early trials, and staff at Ransom Seed Labs (Carpinteria, CA, USA) recommended soaking at higher temperature for a longer period. During trials on intact seed, seed staining rates were 39% (*E. mohavense*) and 61% (*E. wallacei*). We applied these refined staining protocols to a subset of the retained seed pools for each species to differentiate between and subsequently count the total number of retained dead seed (Figure 2c, flow E) and retained live seed (Figure 2c, flow D). We refer to the percentage of stained seed during assays as the seed staining rate (sometimes referred to as percent viability in other studies). Seed bank survival is then calculated by multiplying the percentage of seed remaining in the retained seed pool (Figure 2c, flow C) by the seed staining rate (i.e., the proportion of retained seeds stained during tetrazolium assays; Figure 2c, flow D)—see Supplementary Tables S3 and S4 for seed staining rates and seed bank survival calculations.

We used the total number of seeds collected in all seed packets within a cohort to represent the seed bank (100%). The live seed pool (Figure 2c, see G) can be expressed as the sum of germinated and live seeds, while the dead seed pool (Figure 2c, see H) can be expressed as the sum of decayed seed and retained dead seed—together adding up to 100%. Seed bank survival is the proportion of retained live seed in the seed bank (Figure 2c, flow D). We measured seed bank pools and types for each species—by cohort, year, and microhabitat—and reported them as percentages of the total seed bank (100%). Sankey diagrams are a visualization technique that display proportionally accurate flows within a defined system. Thus, we used Sankey diagrams to visualize proportional differences in seed bank dynamics across species following the first (Figure 4a) and second year of burial (Figure 4b), and differences across species as well as microhabitat after the second year of burial (Figure 4c, Supplementary Figure S3). For each scenario shown in Sankey diagrams, seed bank survival = the seed bank × percent retained seed × percent stained (viable) seed (see Supplementary Tables S2 and S3 for averaged values used in seed bank survival calculations).

### 4.8. Data Analyses

All statistical analyses were performed in R using a combination of generalized linear models with logit link functions, analysis of variance (ANOVA), Mann–Whitney U, and Kruskal–Wallis tests with Dunn’s multiple comparison post-hoc tests (version 1.2.5042, Rstudio, Boston, MA, USA).

We built quasibinomial generalized linear models (GLMs) with logit link functions to evaluate retained seed pools and seed staining rates (version 1.2.5042, Rstudio, Boston, MA, USA). We used the Anova function in the car package [53] to evaluate models and generate Type III *p*-values, and conducted post-hoc tests on estimated marginal means using the emmeans package [87].

In the GLM evaluating the retained seed pool (Figure 2c, see flow C), the proportion of retained seed per packet was the response, and proportions were weighted by the number of seeds recovered from a given microhabitat and plot (combining seed of the same cohort where multiple packets were collected in the same location). Year, species, microhabitat, seed cohort, and all interactions were included as fixed effects. Plot was not included as a blocking effect because blocks were incomplete. Although quasibinomial approaches are recommended to compensate for overdispersion, overdispersion could not be eliminated, so *p*-values should be regarded as approximate [54].

The GLM evaluating seed survival (Figure 2c, see flow D) used stain presence on individual seeds as the response variable (stain present or absent). Fixed effects included year, species, microhabitat, seed cohort, and all interactions. To test for differences in seed bank survival by burial duration (2017-one growing season, 2018-two growing seasons) between the rare and common species (including comparison within the control microhabitat only, Figure 3a, and across all microhabitats), we used a nonparametric Mann–Whitney U test on two medians using ranks of the sample data, as comparative datasets were not normal (e.g., W = 0.52317, *p*-value = 0.00112, Shapiro–Wilk normality test). To test for differences in seed bank survival across microhabitats for both species combined in each burial period (2017-one growing season, 2018-two growing seasons, Figure 3b), we used a Kruskal–Wallis test (with Dunn’s multiple comparison post-hoc test) for the equality of medians, as these datasets were also not normal (Shapiro–Wilk normality test).

## 5. Conclusions

Understanding the survival of seeds underpins our capacity to predict plant population demographics, identify extinction and invasion risks, assess land management and development decisions, and reveal mechanisms driving ancient and modern plant evolution [6,13,51,88]. Overall, our study provides insight into the early life-stage of desert annual plants using a novel seed bank survival framework, which addresses some longstanding challenges in describing and quantifying seed banks.

## Figures and Tables

**Table 1:** Allocation of 2015 and 2016 seed cohorts to seed bank packets by species.

	Species	Seed Cohort	Number of Seed Bank Packets	Number of Seeds Per Packet	Total Seeds
(a)	*E. mohavense*	2015	90	18	1620
		2016	180	9	1620
(b)	*E. wallacei*	2015	90	14	1260
		2016	180	2	360

**Table 2:** Results from quasibinomial generalized linear model evaluating effects of year, species, microhabitat, seed cohort, and all interactions on the retained seed pool. Bold text indicates a significant difference at the *p* ≤ 0.05 level ^1^; italic text indicates a marginally significant difference at the *p* ≤ 0.10 level.

Predictor	Sum Sq.	Df	*F*-Value	*p*-Value
year	190.94	1	108.77	<0.001
species	52.38	1	29.84	<0.001
microhabitat	17.94	2	5.11	0.007
seed cohort	3.88	1	2.21	0.139
year × species	63.07	1	35.92	<0.001
year × microhabitat	20.17	2	5.74	0.004
species × microhabitat	1.86	2	0.53	0.589
year × seed cohort	0.30	1	0.17	0.681
species × seed cohort	29.83	1	16.99	<0.001
microhabitat × seed cohort	0.55	2	0.16	0.855
year × species × microhabitat	2.76	2	0.79	0.457
year × species × seed cohort	0.05	1	0.03	0.869
year × microhabitat × seed cohort	0.70	2	0.20	0.819
species × microhabitat × seed cohort	0.02	2	0.01	0.994
year × species × microhabitat × seed cohort	0.38	2	0.11	0.897
residuals	330.04	188	NA	NA

^1^ Analysis of deviance table reports Type III tests with error estimates based on Pearson residuals generated by the Anova function in the car package [53]. The quasibinomial approach did not eliminate overdispersion from models so *p*-values should be regarded as approximate [54].

**Table 3:** Results from quasibinomial generalized linear models evaluating effects of year, species, microhabitat, seed cohort, and all interactions on results of stain-based assays (i.e., % of seeds from the retained seed pool that stained red). Bold text indicates a significant difference at the *p* ≤ 0.05 level ^1^; italic text indicates a marginally significant difference at the *p* ≤ 0.10 level.

Predictor	Sum Sq.	Df	*F*-Value	*p*-Value
Year	2.97	1	2.90	0.089
species	41.99	1	41.00	<0.001
microhabitat	0.00	2	0.00	1.000
seed cohort	2.02	1	1.97	0.161
year × species	11.48	1	11.21	0.001
year × microhabitat	0.00	2	0.00	1.000
species × microhabitat	0.00	2	0.00	1.000
year × seed cohort	4.55	1	4.45	0.035
species × seed cohort	0.87	1	0.85	0.357
microhabitat × seed cohort	0.00	2	0.00	1.000
year × species × microhabitat	0.00	2	0.00	1.000
year × species × seed cohort	7.95	1	7.76	0.006
year × microhabitat × seed cohort	0.00	2	0.00	1.000
species × microhabitat × seed cohort	0.00	2	0.00	1.000
year × species × microhabitat × seed cohort	0.00	2	0.00	1.000
residuals	595.00	581	NA	NA

^1^ Analysis of deviance table reports Type III tests with error estimates based on Pearson residuals generated by the Anova function in the car package [53]. The quasibinomial approach did not eliminate overdispersion from the model so *p*-values should be regarded as approximate [54].

## Supplementary Materials

The following are available online at https://www.mdpi.com/2223-7747/9/9/1125/s1, Figure S1. The retained seed pool from seed bank packets collected in 2017 and 2018. Figure S2. Seed staining rate (%) for the subsets of retained seed from packets collected in 2017 and 2018; percentages of stained *E. mohavense* seed are shown for the 2015 seed cohort and the 2016 seed cohort. Figure S3. The seed bank survival model showing empirical seed bank pools and types in the Control and Shade microhabitats for (**a**) *E. mohavense* and (**b**) *E. wallacei* (averaged across cohorts for each species) after two years of burial. Table S1. Sample sizes for packets recovered at the (**a**) Caliche Pan (*E. mohavense*), and (**b**) Gravelly Bajada (*E. wallacei*) site. Table S2. Average retained seed pool for each species broken down by year of packet collection, seed cohort, and microhabitat. Table S3. Average seed staining rates for each species broken down by year of packet collection, seed cohort, and microhabitat. Table S4. Retained seed pools, seed staining rates, and calculated seed bank survival (%) from field data.

## References

[1] Sandel B., Gutiérrez A.G., Reich P.B., Schrodt F., Dickie J., Kattge J. (2015). Estimating the missing species bias in plant trait measurements. J. Veg. Sci..

[2] Saatkamp A., Cochrane A., Commander L., Guja L.K., Jimenez-Alfaro B., Larson J., Nicotra A., Poschlod P., Silveira F.A.O., Cross A.T. (2019). A research agenda for seed-trait functional ecology. New Phytol..

[3] Wisheu I.C., Keddy P.A. (1991). Seed banks of a rare wetland plant community: Distribution patterns and effects of human-induced disturbance. J. Veg. Sci..

[4] Moriuchi K.S., Venable D.L., Pake C.E., Lange T. (2000). Direct measurement of the seed bank age structure of a Sonoran Desert annual plant. Ecology.

[5] Saatkamp A., Poschlod P., Venable D.L. (2014). The functional role of soil seed banks in natural communities. Seeds: The Ecology of Regeneration in Plant Communities.

[6] LaForgia M.L., Spasojevic M.J., Case E.J., Latimer A.M., Harrison S.P. (2018). Seed banks of native forbs, but not exotic grasses, increase during extreme drought. Ecology.

[7] Cochrane J.A. (2020). Thermal requirements underpinning germination allude to risk of species decline from climate warming. Plants.

[8] Ge X.Y.M., Scholl J.P., Basinger U., Huxman T.E., Venable D.L. (2019). Functional trait trade-off and species abundance: Insights from a multi-decadal study. Ecol. Lett..

[9] Tielbörger K., Prasse R. (2009). Do seeds sense each other? Testing for density-dependent germination in desert perennial plants. Oikos.

[10] Sartor C.E., Marone L. (2010). A plurality of causal mechanisms explains the persistence or transience of soil seed banks. J. Arid Environ..

[11] Rehbein J.A., Watson J.E.M., Lane J.L., Sonter L.J., Venter O., Atkinson S.C., Allan J.R. (2020). Renewable energy development threatens many globally important biodiversity areas. Glob. Chang. Biol..

[12] Hernandez R.R., Hoffacker M.K., Murphy-Mariscal M.L., Wu G., Allen M.F. (2015). Solar energy development impacts on land cover change and protected areas. Proc. Natl. Acad. Sci. USA.

[13] Moore-O’Leary K.A., Hernandez R.R., Johnston D.S., Abella S.R., Tanner K.E., Swanson A.C., Kreitler J., Lovich J.E. (2017). Sustainability of utility-scale solar energy—Critical ecological concepts. Front. Ecol. Environ..

[14] Holmes P.M., Newton R.J. (2004). Patterns of seed persistence in South African fynbos. Plant Ecol..

[15] Ooi M.K.J., Auld T.D., Whelan R.J. (2007). Distinguishing between persistence and dormancy in soil seed banks of three shrub species from fire-prone southeastern Australia. J. Veg. Sci..

[16] Auld T.D., Keith D.A., Bradstock R.A. (2000). Patterns in longevity of soil seedbanks in fire-prone communities of south-eastern Australia. Aust. J. Bot..

[17] Sotomayor D.A., Gutiérrez J.R. (2015). Seed bank of desert annual plants along an aridity gradient in the southern Atacama coastal desert. J. Veg. Sci..

[18] Caballero I., Olano J.M., Loidi J., Escudero A. (2003). Seed bank structure along a semi-arid gypsum gradient in Central Spain. J. Arid Environ..

[19] Van Mourik T.A., Stomph T.J., Murdoch A.J. (2005). Why high seed densities within buried mesh bags may overestimate depletion rates of soil seed banks. J. Appl. Ecol..

[20] Pakeman R.J., Small J.L., Torvell L. (2012). Edaphic factors influence the longevity of seeds in the soil. Plant Ecol..

[21] Rivera D., Jáuregui B.M., Peco B. (2012). The fate of herbaceous seeds during topsoil stockpiling: Restoration potential of seed banks. Ecol. Eng..

[22] Reichman O.J. (1984). Spatial and Temporal Variation of Seed Distributions in Sonoran Desert Soils. J. Biogeogr..

[23] Kemp P.R. (1989). Seed Banks and Vegetation Processes in Deserts. Ecology of Soil Seed Banks.

[24] Cabin R.J., Marshall D.L., Mitchell R.J. (2000). The demographic role of soil seed banks. II. Investigations of the fate of experimental seeds of the desert mustard Lesquerella fendleri. J. Ecol..

[25] Angert A.L., Huxman T.E., Barron-Gafford G.A., Gerst K.L., Venable D.L. (2007). Linking growth strategies to long-term population dynamics in a guild of desert annuals. J. Ecol..

[26] Huxman T.E., Kimball S., Angert A.L., Gremer J.R., Barron-Gafford G.A., Lawrence Venable D. (2013). Understanding past, contemporary, and future dynamics of plants, populations, and communities using Sonoran desert winter annuals. Am. J. Bot..

[27] Violle C., Borgy B., Choler P. (2015). Trait databases: Misuses and precautions. J. Veg. Sci..

[28] Saatkamp A., Affre L., Baumberger T., Dumas P.J., Gasmi A., Gachet S., Arène F. (2011). Soil depth detection by seeds and diurnally fluctuating temperatures: Different dynamics in 10 annual plants. Plant Soil.

[29] Baskin J.M., Baskin C.C. (1976). High temperature requirements for afterripening in seeds of winter annuals. New Phytol..

[30] Li Y.M., Shaffer J.P., Hall B., Ko H. (2019). Soil-borne fungi influence seed germination and mortality, with implications for coexistence of desert winter annual plants. PLoS ONE.

[31] Brown J.H., Reichman O.J., Davidson D.W. (1979). Granivory in desert ecosystems. Annu. Rev. Ecol. Syst..

[32] Saatkamp A., Affre L., Dutoit T., Poschlod P. (2009). The seed bank longevity index revisited: Limited reliability evident from a burial experiment and database analyses. Ann. Bot..

[33] Thompson K. (2009). The functional ecology of soil seed banks. Seeds: The Ecology of Regeneration in Plant Communities.

[34] Adondakis S., Venable D.L. (2004). Dormancy and germination in a guild of Sonoran Desert annuals. Ecology.

[35] Parker V.T., Simpson R.L., Leck M.A. (1989). Pattern and Process in the Dynamics of Seed Banks. Ecology of Soil Seed Banks.

[36] Lyons K.G., Brigham C.A., Traut B.H., Schwartz M.W. (2005). Rare species and ecosystem functioning. Conserv. Biol..

[37] Lavergne S., Garnier E., Debussche M. (2003). Do rock endemic and widespread plant species differ under the Leaf-Height-Seed plant ecology strategy scheme?. Ecol. Lett..

[38] Long R.L., Gorecki M.J., Renton M., Scott J.K., Colville L., Goggin D.E., Commander L.E., Westcott D.A., Cherry H., Finch-Savage W.E. (2015). The ecophysiology of seed persistence: A mechanistic view of the journey to germination or demise. Biol. Rev..

[39] Tanner K.E., Moore-O’Leary K., Parker I.M., Pavlik B.M., Hernandez R.R. Microhabitats associated with solar energy development alter demography of two desert annuals.

[40] Hernandez R.R., Hoffacker M.K., Field C.B. (2015). Efficient use of land to meet sustainable energy needs. Nat. Clim. Chang..

[41] Hernandez R.R., Hoffacker M.K., Murphy-Mariscal M.L., Wu G.C., Allen M.F. (2017). Land-sparing opportunities for solar energy development in agricultural landscapes: A case study of the Central Valley. Environ. Sci. Technol..

[42] Hernandez R.R., Armstrong A., Burney J., Ryan G., Moore-O’Leary K., Diédhiou I., Grodsky S.M., Saul-Gershenz L., Davis R., Macknick J. (2019). Techno–ecological synergies of solar energy for global sustainability. Nat. Sustain..

[43] Parker S.S., Cohen B.S., Moore J. (2018). Impact of solar and wind development on conservation values in the Mojave Desert. PLoS ONE.

[44] Grodsky S.M., Leary K.A.M., Hernandez R.R. From butterflies to bighorns: Multi-dimensional species-species and species-process interactions may inform sustainable solar energy development in desert ecosystems. Proceedings of the 31st Annual Desert Symposium, California State University Desert Studies Center.

[45] Grodsky S.M., Hernandez R.R. (2020). Reduced ecosystem services of desert plants from ground-mounted solar energy. Nat. Sustain..

[46] Haegel N.M., Atwater H., Barnes T., Breyer C., Burrell A., Chiang Y.-M., De Wolf S., Dimmler B., Feldman D., Glunz S. (2019). Terawatt-scale photovoltaics: Transform global energy. Science..

[47] Tanner K.E., Moore-O’Leary K.A., Parker I.M., Pavlik B.M., Hernandez R.R. (2020). Simulated solar panels create altered microhabitats in desert landforms. Ecosphere.

[48] Cohen D. (1966). Optimizing reproduction in a randomly varying environment. J. Theor. Biol..

[49] Salguero-Gómez R., Siewert W., Casper B.B., Tielbörger K. (2012). A demographic approach to study effects of climate change in desert plants. Philos. Trans. R. Soc. B Biol. Sci..

[50] Jiménez-Alfaro B., Silveira F.A.O., Fidelis A., Poschlod P., Commander L.E. (2016). Seed germination traits can contribute better to plant community ecology. J. Veg. Sci..

[51] Chiquoine L.P., Abella S.R. (2018). Soil seed bank assay methods influence interpretation of non-native plant management. Appl. Veg. Sci..

[52] Walck J.L., Baskin J.M., Baskin C.C., Hidayati S.N. (2005). Defining transient and persistent seed banks in species with pronounced seasonal dormancy and germination patterns. Seed Sci. Res..

[53] Fox J., Weisbery S. (2018). An R Companion to Applied Regression.

[54] Carruthers E., Lewis K., Mccue T., Westley P. (2008). Generalized Linear Models: Model Selection, Diagnostics, and Overdispersion.

[55] Kalisz S., McPeek M.A. (1992). Demography of an age-structured annual: Resampled projection matrices, elasticity analyses, and seed bank effects. Ecology.

[56] Pluntz M., Le Coz S., Peyrard N., Pradel R., Choquet R., Cheptou P.O. (2018). A general method for estimating seed dormancy and colonisation in annual plants from the observation of existing flora. Ecol. Lett..

[57] Tevis L. (1958). A population of desert Ephemerals germinated by less than one inch of rain. Ecology.

[58] Beatley J.C. (1974). Phenological events and their environmental triggers in Mojave Desert ecosystems. Ecology.

[59] Filazzola A., Lortie C.J. (2014). A systematic review and conceptual framework for the mechanistic pathways of nurse plants. Glob. Ecol. Biogeogr..

[60] Kos M., Poschlod P. (2007). Seeds use temperature cues to ensure germination under nurse-plant shade in Xeric Kalahari Savannah. Ann. Bot..

[61] Macknick J., Beatty B., Hill G. (2013). Overview of Opportunities for Co-Location of Solar Energy Technologies and Vegetation.

[62] Yang Y., Hobbie S.E., Hernandez R.R., Fargione J., Grodsky S.M., Tilman D., Zhu Y.-G., Luo Y., Smith T.M., Jungers J.M. (2020). Restoring Abandoned Farmland to Mitigate Climate Change on A Full Earth. One Earth.

[63] Schafer M., Kotanen P.M. (2003). The influence of soil moisture on losses of buried seeds to fungi. Acta Oecologica.

[64] Mordecai E.A. (2012). Soil moisture and fungi affect seed survival in California Grassland annual plants. PLoS ONE.

[65] Tanner K.E. (2020). Plant Response to Land Use Change in Two Iconically Stressful Habitats: California’s Desert Solar Fields and Restored Coastal Salt Marshes.

[66] Cohen D. (1967). Optimizing reproduction in a randomly varying environment when a correlation may exist between the conditions at the time a choice has to be made and the subsequent outcome. J. Theor. Biol..

[67] Grime J.P., Mason G., Curtis A.V., Rodman J., Band S.R. (1981). A Comparative Study of Germination Characteristics in a Local Flora. J. Ecol..

[68] Keddy P.A. (1992). Assembly and response rules: Two goals for predictive community ecology. J. Veg. Sci..

[69] Leck M.A. (1989). Ecology of Soil Seed Banks.

[70] Del Castillo R.F. (1994). Factors influencing the genetic structure of Phacelia dubia, a species with a seed bank and large fluctuations in population size. Heredity.

[71] Venable D.L., Kimball S. (2012). Population and community dynamics in variable environments: The desert annual system. Temporal Dynamics and Ecological Process.

[72] Fenner M. (1991). The effects of the parent environment on seed germinability. Seed Sci. Res..

[73] Philippi T. (1993). Bet-hedging germination of desert annuals: Variation among populations and maternal effects in *Lepidium lasiocarpum*. Am. Nat..

[74] Schlesinger W.H., Jones C.S. (1984). The comparative importance of overland runoff and mean annual rainfall to shrub communities of the Mojave Desert. Bot. Gaz..

[75] Hamerlynck E., Huxman T.E., McAuliffe J.R., Smith S.D. (2004). Carbon isotope discrimination and foliar nutrient status of *Larrea tridentata* (creosote bush) in contrasting Mojave Desert soils. Oecologia.

[76] Vandergast A.G., Inman R.D., Barr K.R., Nussear K.E., Esque T.C., Hathaway S.A., Wood D.A., Medica P.A., Breinholt J.W., Stephen C.L. (2013). Evolutionary Hotspots in the Mojave Desert. Diversity.

[77] Thorne J.H., Viers J.H., Price J., Stoms D.M. (2009). Spatial Patterns of Endemic Plants in California. Nat. Areas J..

[78] Baldwin B., Goldman D.H., Keil D.J., Patterson R., Rosatti T.J. (2012). The Jepson Manual: Vascular Plants of California.

[79] California Native Plant Society: Calscape-Barstow Woolly Sunflower. https://calscape.org/Eriophyllum-mohavense-().

[80] Mooring J.S. (2002). Experimental hybridizations of *Eriophyllum annuals* (Asteraceae, Helenieae). Am. J. Bot..

[81] Consortium of California Herbaria The Calflora Database: Eriophyllum mohavense. https://www.calflora.org/cgi-bin/species_query.cgi?where-calrecnum=3439.

[82] State of California Desert Renewable Energy Conservation Plan. https://www.energy.ca.gov/programs-and-topics/programs/desert-renewable-energy-conservation-plan.

[83] Colville L., Pritchard H.W. (2019). Seed life span and food security. New Phytol..

[84] Freas K.E., Kemp P.R. (1983). Some relationships between environmental reliability and seed dormancy in desert annual plants. J. Ecol..

[85] Porter R.H., Durrell M., Romm H.J. (1947). The use of 2,3,5-Triphenyl-tetrazoliumchloride as a measure of seed germinability. Plant Physiol..

[86] Pake C.E., Venable D.L. (1996). Seed banks in desert annuals: Implications for persistence and coexistence in variable environments. Ecology.

[87] Lenth R., Singmann H., Love J., Buerkner P., Herve M. (2019). Emmeans: Estimated Marginal Means, aka Least-Squares Means, Version 1.3.4; R Package. https://CRAN.R-project.org/package=emmeans.

[88] Shoemaker W.R., Lennon J.T. (2018). Evolution with a seed bank: The population genetic consequences of microbial dormancy. Evol. Appl..

