# Supplementary material for: Simulated Photovoltaic Solar Panels Alter the Seed Bank Survival of Two Desert Annual Plant Species
Source: Plants (Basel). 2020 Aug 31;9(9):1125. doi: 10.3390/plants9091125 (PMC7570262; doi:10.3390/plants9091125)
Supplement: Supplementary file 1 [file plants-09-01125-s001.pdf]

plants

Simulated photovoltaic solar panels alters the seed bank survival of desert annual plant species

Supporting Information

Rebecca R. Hernandez[1,2*], Karen E. Tanner[3], Sophia Haji[3], Ingrid M. Parker[3], Bruce M. Pavlik[4], and Kara A. Moore-O'Leary[5]

1 Department of Land, Air & Water Resources, University of California, Davis, One Shields Avenue, Davis, CA 95616
2 Wild Energy Initiative, John Muir Institute of the Environment, University of California, Davis, One Shields Avenue, Davis, CA 95616
3 Ecology and Evolutionary Biology Department, University of California, Santa Cruz, 1156 High St, Santa Cruz, CA 95064
4 Conservation Department, Red Butte Garden and Arboretum, University of Utah, Salt Lake City, UT 84108
5 U.S. Fish and Wildlife Service, Pacific Southwest Region, 3020 State University Drive East, Sacramento, CA 95819
* Correspondence: rrhernandez@ucdavis.edu

Received: date; Accepted: date; Published: date

Figure S1. The retained seed pool from seed bank packets collected in 2017 and 2018.

Figure S2. Seed staining rate (%) for the subsets of retained seed from packets collected in 2017 and 2018; percentages of stained *E. mohavense* seed are shown for the 2015 seed cohort and the 2016 seed cohort.

Figure S3. The seed bank survival model showing empirical seed bank pools and types in the Control and Shade microhabitats for (a) *E. mohavense* and (b) *E. wallacei* (averaged across cohorts for each species) after two years of burial.

Table S1. Sample sizes for packets recovered at the (a) Caliche Pan (*E. mohavense*), and (b) Gravelly Bajada (*E. wallacei*) site.

Table S2. Average retained seed pool for each species broken down by year of packet collection, seed cohort, and microhabitat.

Table S3. Average seed staining rates for each species broken down by year of packet collection, seed cohort, and microhabitat.

Table S4. Retained seed pools, seed staining rates, and calculated seed bank survival (%) from field data.

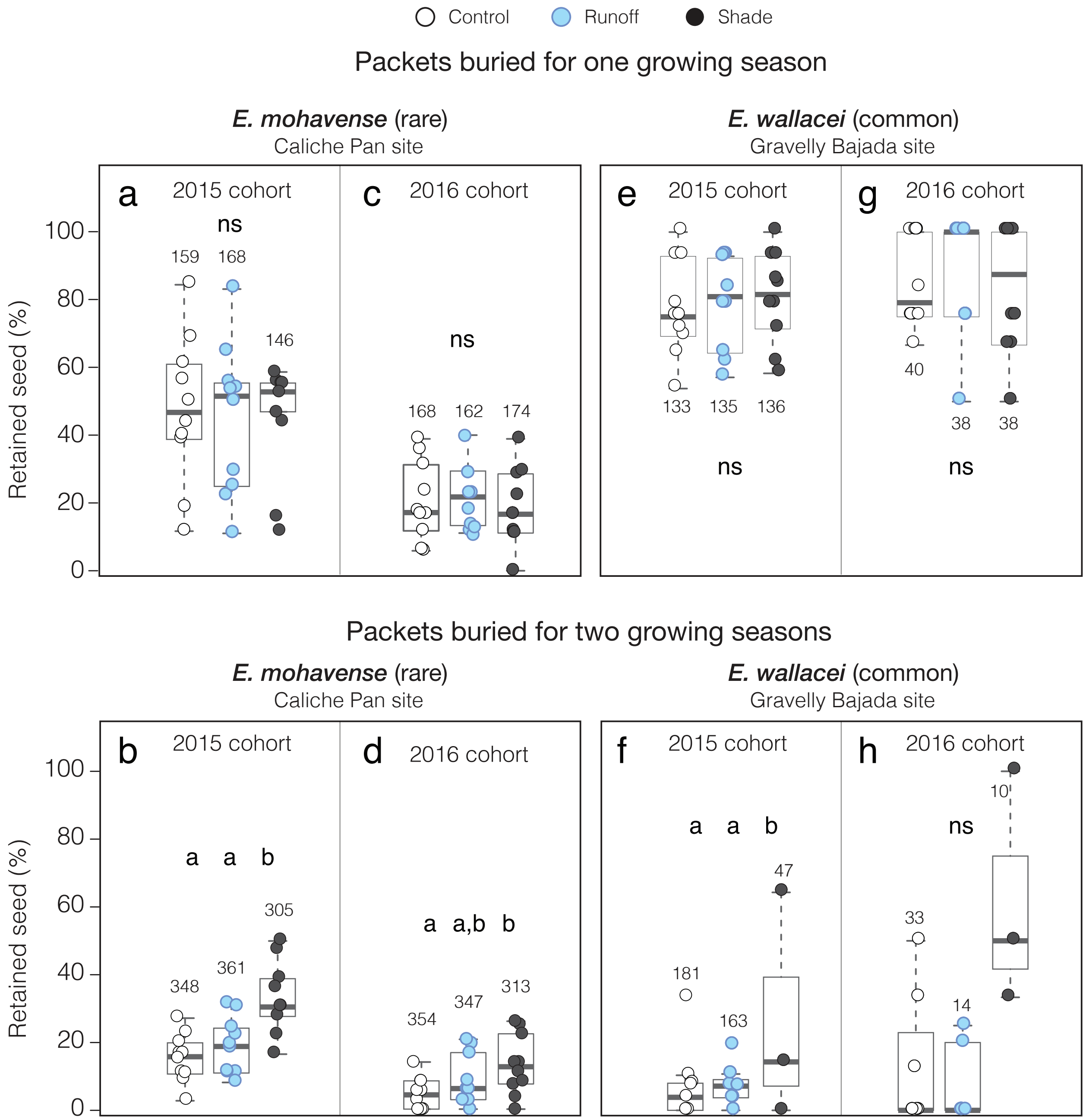

Figure S1. The retained seed pool from seed bank packets collected in 2017 (top row) and 2018 (bottom row). Percentages of retained, intact *E. mohavense* seed are shown in (a, b) for the 2015 seed cohort, and (c, d) for the 2016 seed cohort. Percentages of retained *E. wallacei* seed are shown in (e, f) for the 2015 seed cohort, and (g, h) for the 2016 seed cohort. Data points overlaid on boxplots show the number of packets collected from each microhabitat, and the numbers above each boxplot show the total number of seeds recovered from collected packets. Where letters above boxplots differ, the percentages of retained seed recovered were significantly different at the $p < 0.05$ level. Retained seed pools broken down by species, cohort, and microhabitat are provided in Table S2.

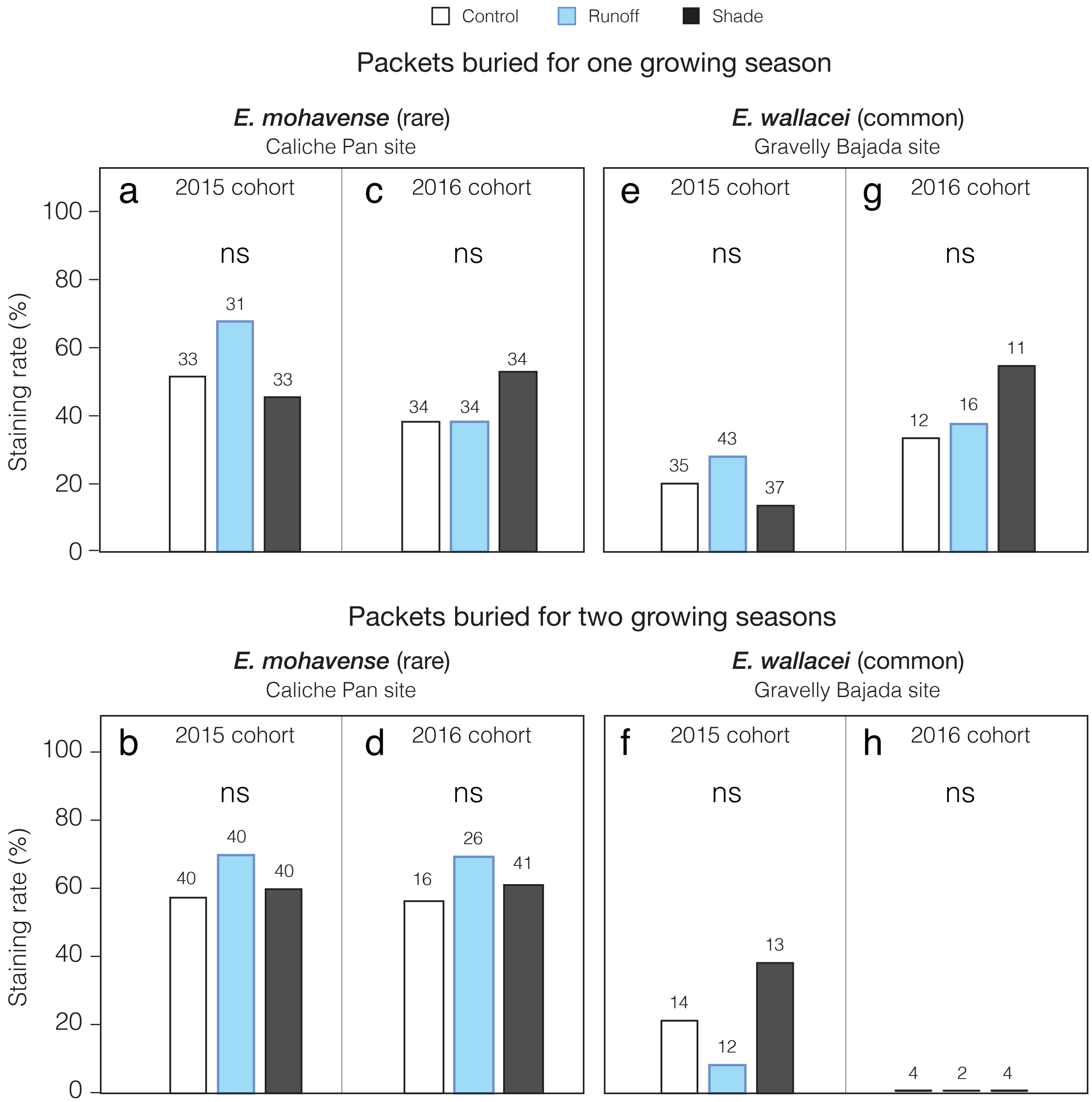

Figure S2. Staining rate (%) for the subsets of retained seed from packets collected in 2017 (top row) and 2018 (bottom row); percentages of stained *E. mohavense* seed are shown in (a, b) for the 2015 seed cohort, and (c, d) for the 2016 seed cohort. Percentages of stained *E. wallacei* seed are shown in (e, f) for the 2015 seed cohort and (g, h) for the 2016 seed cohort. Numbers above bar plots represent the total number of intact seeds subjected to tetrazolium assays. Final seed bank survival (%) is calculated by multiplying the retained seed pool by the proportion (i.e., decimal form of the percent) of the staining rate (see Supplementary Information, Table S3 for full seed bank survival calculations).

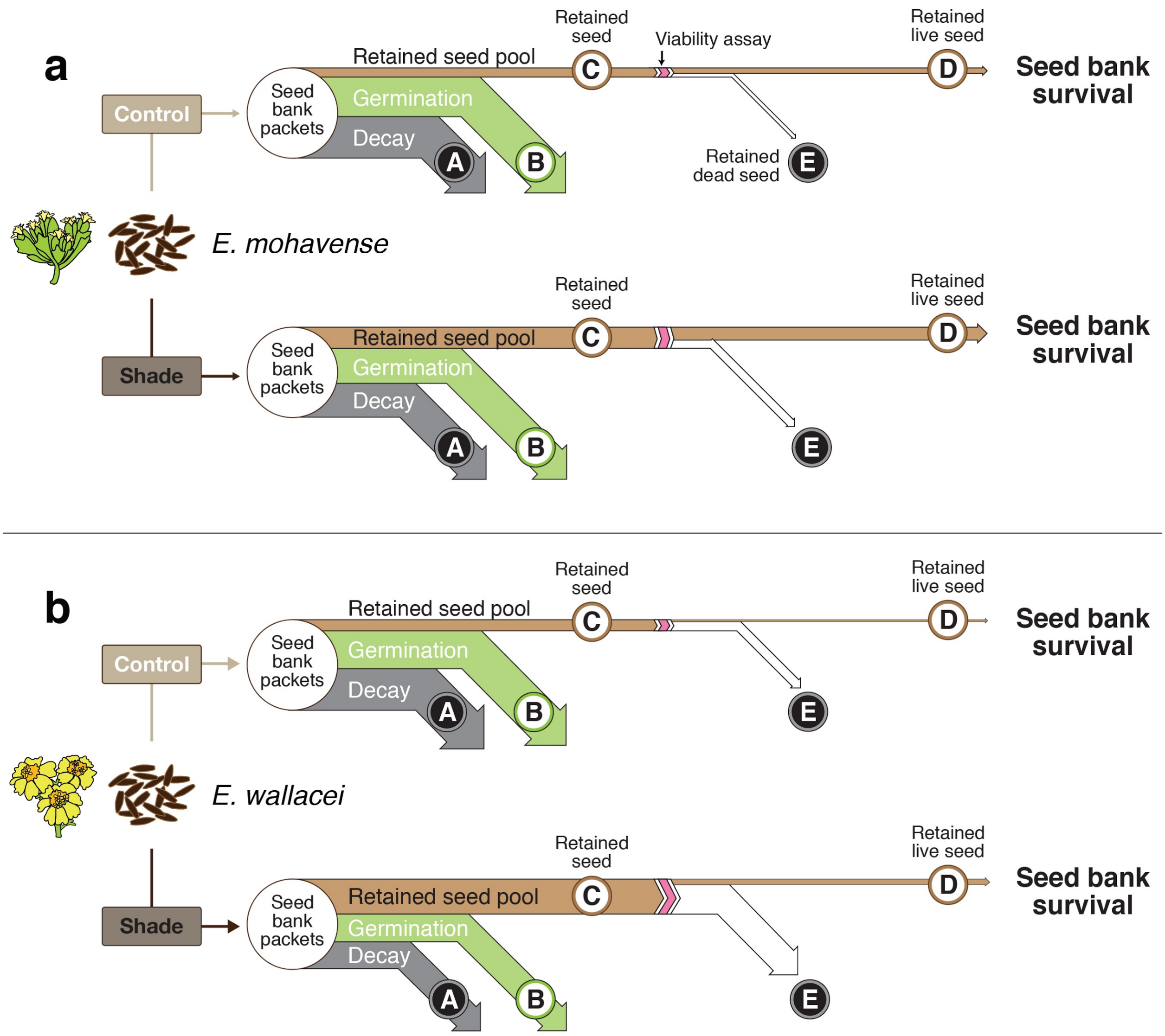

Figure S3. The seed bank survival model showing empirical seed bank pools and types in the Control and Shade microhabitats for (a) *E. mohavense* and (b) *E. wallacei* (averaged across cohorts for each species) after two years of burial. We observed higher seed retention in the Shade compared to the other two microhabitats (we show only Shade and Control flows here; flows in the Runoff microhabitat are very similar to Control flows). We cannot confidently partition decayed seed (flow A) from germinated seed (flow B) in the expended seed pool (due to the delay between the winter annual germination period and collection of packets in spring), so we visualize these flows as equivalent in size. Flows exiting the staining assay (pink chevron) visualize the percentage of live seed for a subset of the retained seed pools (C) exposed to staining assays.

Table S1. Sample sizes for packets recovered at the (a) Caliche Pan (*E. mohavense*) and (b) Gravelly Bajada (*E. wallacei*) site.

	Year collected	Seed cohort	Microhabitat	Total seeds	Total packets
**(a)** ***E. mohavense***					
	2017	2015	Control	159	10
			Runoff	168	10
			Shade	146	9
		2016	Control	168	19
			Runoff	162	20
			Shade	174	20
	2018	2015	Control	348	20
			Runoff	361	20
			Shade	305	17
		2016	Control	354	40
			Runoff	347	40
			Shade	313	34
**(b)** ***E. wallacei***					
	2017	2015	Control	133	10
			Runoff	135	10
			Shade	136	10
		2016	Control	40	21
			Runoff	38	20
			Shade	38	20
	2018	2015	Control	181	14
			Runoff	163	12
			Shade	47	4
		2016	Control	33	18
			Runoff	14	8
			Shade	10	5

Table S2. Average retained seed pool for each species broken down by year of packet collection, seed cohort, and microhabitat. Rows where packets were collected at less than 10 plots indicate a loss of packets in the field. Rabbits were observed digging around buried packets and were the likely culprits of their disappearance (Tanner, *pers. observ.*). Most packets were lost in the second year at the Gravelly Bajada (*E. wallacei*) site.

Species	Year packets collected	Seed cohort	Microhabitat	Number of plots	Number of seeds recovered	Retained seed pool	Retained seed (%)
**a) *E. mohavense***	2017	2015	Control	10	159	74	0.47
			Runoff	10	168	75	0.45
			Shade	9	146	65	0.45
		2016	Control	10	168	34	0.20
			Runoff	10	162	35	0.22
			Shade	10	174	32	0.18
	2018	2015	Control	10	348	53	0.15
			Runoff	10	361	68	0.19
			Shade	9	305	99	0.32
		2016	Control	10	354	16	0.05
			Runoff	10	347	30	0.09
			Shade	10	313	44	0.14
**b) *E. wallacei***	2017	2015	Control	10	133	103	0.77
			Runoff	10	135	107	0.79
			Shade	10	136	110	0.81
		2016	Control	10	40	34	0.85
			Runoff	10	38	34	0.89
			Shade	10	38	32	0.84
	2018	2015	Control	9	181	17	0.09
			Runoff	7	163	13	0.08
			Shade	3	47	13	0.28
		2016	Control	7	33	5	0.15
			Runoff	5	14	2	0.14
			Shade	3	10	5	0.50

Table S3. Average seed staining rates for each species broken down by year of packet collection, seed cohort, and microhabitat.

	Species	Year packets collected	Seed Cohort	Microhabitat	Number of seeds assayed	Retained live seed pool	Staining rate
a)	*E. mohavense*	2017	2015	Control	33	17	0.52
				Runoff	31	21	0.68
				Shade	33	15	0.45
			2016	Control	34	13	0.38
				Runoff	34	13	0.38
				Shade	34	18	0.53
		2018	2015	Control	40	23	0.58
				Runoff	40	28	0.70
				Shade	40	24	0.60
			2016	Control	16	9	0.56
				Runoff	26	18	0.69
				Shade	41	25	0.61
b)	*E. wallacei*	2017	2015	Control	35	7	0.20
				Runoff	43	12	0.28
				Shade	37	5	0.14
			2016	Control	12	4	0.33
				Runoff	16	6	0.38
				Shade	11	6	0.55
		2018	2015	Control	14	3	0.21
				Runoff	12	1	0.08
				Shade	13	5	0.38
			2016	Control	4	0	0
				Runoff	2	0	0
				Shade	4	0	0

Table S4. Retained seed pools, staining rates, and seed bank survival (%, calculated using Equation 1) from field data. (a) Empirical values by year and species (averaged across cohorts and microhabitats); (b) empirical values by year and microhabitat (averaged across species and cohorts). Retained seed pools and seed staining rates broken down by species, year, cohort, and microhabitat are provided in Tables S2 and S3.

	Year collected	Retained seed pool	Staining rate	Seed bank survival
**a) By species, all microhabitats combined**				
E. mohavense	2017	32.7%	49.0%	16.7%
E. wallacei	2017	82.7%	31.1%	26.1%
E. mohavense	2018	15.6%	62.3%	9.8%
E. wallacei	2018	20.7%	11.4%	2.2%
**b) By microhabitat, both species combined**				
Control	2017	57.3%	35.8%	18.9%
Runoff	2017	58.7%	42.8%	23.5%
Shade	2017	57.0%	41.6%	21.7%
Control	2018	11.1%	33.8%	3.3%
Runoff	2018	12.4%	36.9%	5.0%
Shade	2018	31.0%	39.9%	9.7%
